# Short-Term Responses of Air Quality to Changes in Emissions under the Representative Concentration Pathway 4.5 Scenario over Brazil

**DOI:** 10.3390/atmos11080799

**Published:** 2020-07-29

**Authors:** Daniel Schuch, Maria de Fatima Andrade, Yang Zhang, Edmilson Dias de Freitas, Michelle L. Bell

**Affiliations:** 1 Department of Civil and Environmental Engineering, Northeastern University, Boston, MA 02115, USA; 2 Instituto de Astronomia, Geofísica e Ciências Atmosféricas, Universidade de São Paulo, São Paulo 05508-090, Brazil; 3 School of Forestry & Environmental Studies, Yale University, New Haven, CT 06511, USA

**Keywords:** air quality responses, atmospheric emissions, climate change, WRF-Chem

## Abstract

Brazil, one of the world’s fastest-growing economies, is the fifth most populous country and is experiencing accelerated urbanization. This combination of factors causes an increase in urban population that is exposed to poor air quality, leading to public health burdens. In this work, the Weather Research and Forecasting Model with Chemistry is applied to simulate air quality over Brazil for a short time period under three future emission scenarios, including current legislation (CLE), mitigation scenario (MIT), and maximum feasible reduction (MFR) under the Representative Concentration Pathway 4.5 (RCP4.5), which is a climate change scenario under which radiative forcing of greenhouse gases (GHGs) reach 4.5 W m^−2^ by 2100. The main objective of this study is to determine the sensitivity of the concentrations of ozone (O_3_) and particulate matter with aerodynamic diameter 2.5 µm or less (PM_2.5_) to changes in emissions under these emission scenarios and to determine the signal and spatial patterns of these changes for Brazil. The model is evaluated with observations and shows reasonably good agreement. The MFR scenario leads to a reduction of 3% and 75% for O_3_ and PM_2.5_ respectively, considering the average of grid cells within Brazil, whereas the CLE scenario leads to an increase of 1% and 11% for O_3_ and PM_2.5_ respectively, concentrated near urban centers. These results indicate that of the three emission control scenarios, the CLE leads to poor air quality, while the MFR scenario leads to the maximum improvement in air quality. To the best of our knowledge, this work is the first to investigate the responses of air quality to changes in emissions under these emission scenarios for Brazil. The results shed light on the linkage between changes of emissions and air quality.

## 1 Introduction

Brazil is the largest country of South America, both in area and population, with 208 million inhabitants in an area of 8 million km^2^. Brazil also contains the biggest tropical forest in the world, the Amazon Forest, which covers an area of 3.3 million km^2^ (60% of the forest area). The main sources of air pollutant emissions are the vehicular fleet, biomass burning, and industrial processes. The burning of agricultural residues and natural vegetation occurs frequently in rural areas in central and northern parts of Brazil. Air pollution and climate change are critical environmental risks for the country and elsewhere. According to the World Health Organization (WHO), 91% of the world’s population does not breathe clean air, and more than half of the urban population is exposed to levels of ambient pollution at least 2.5 times above the health-based guidelines [1]. Air pollution is associated with a broad spectrum of acute and chronic illnesses. It is estimated to cause about 16% of lung cancer deaths, 25% of chronic obstructive pulmonary disease deaths, 44% of all deaths from cardiovascular diseases (about 17% of ischemic heart disease and stroke), and about 26% of respiratory infection deaths over the world [1].

Particulate matter with an aerodynamic diameter of 2.5 µm or less (PM_2.5_) and tropospheric ozone (O_3_) are two of the atmospheric pollutants that cause adverse health impacts, as is extensively documented in the literature [2–5]. PM_2.5_ originates from a large number of sources and atmospheric processes. Primary PM_2.5_ is directly emitted by a variety of sources with anthropogenic origin, such as agricultural operations, industrial processes, combustion of wood and fossil fuels, construction and demolition activities, and vehicular resuspension of road dust. It can also be emitted by natural sources, such as wildfires, and mechanical processes, such as dust resuspension, wear, fragmentation of vehicle brakes and tires, and suspension of biological matter. Secondary PM_2.5_ is produced by the chemical conversion of gaseous precursors in the atmosphere into condensable compounds (e.g., sulfate, nitrate, ammonium, secondary organic matter) and subsequent partitioning in the particulate phase. Homogeneous nucleation processes initiate the formation of new particles from the gas phase that can grow by condensation of condensable vapor molecules [6]. PM_2.5_ is also formed from both primary and secondary ultrafine particles (defined as suspended particles with an aerodynamic diameter of 1.0 µm or less) that grow via atmospheric condensation of anthropogenic and biogenic organic compounds, sulfuric acid, and nitrates on the surface of particles. O_3_ is a secondary gaseous pollutant formed by the photolytic reaction of nitrogen oxides and the photochemical oxidation of volatile organic compounds (VOCs) and carbon monoxide by hydroxyl radicals. In polluted regions with large sources of NO_x_ and VOCs, high concentrations of O_3_ in the near-surface air represent major air pollution problems [7]. These pollutants have moderate atmospheric lifetimes (from hours to days) and hence, inhomogeneous atmospheric distributions, presenting a close link between net ozone change and radiative forcing [8], but also influenced by background concentration from hemispheric scales to the local scale [9].

PM may also be affected by changes in climate and emissions. PM is anticipated to decrease by 2% to 18% by 2050 due to the increase of wet deposition associated with the increase of precipitation in the U.S. [10]. Simulations on air quality over Portugal using climate and emissions projections for 2050 found that the NO_2_ annual mean will decrease by 50% and PM_10_ will increase by 13% [11]. However, increases and extremes in PM_10_ and O_3_ levels are estimated to occur more often, displaying a higher frequency of daily exceedances of legislated annual standards. The authors attributed the air quality degradation to future climate trends, which will cause warmer and dryer weather conditions. A study on the potential impact of climate changes on wildfires and the resulting air pollution for the western U.S. found that Northern California, Western Oregon, and the Great Plains are likely to suffer the highest exposure to wildfire smoke in the future [12]. These results pointed to the potential health impacts of increasing wildfire smoke and the need to establish or modify U.S. wildfire management and evacuation programs at high-risk regions and for high-risk populations. Evaluation of the impact of climate change and future emissions scenarios on O_3_ and PM_2.5_ over India showed an increase of 4% in the northern region and a decrease of 3% in the southern region caused by changes in biogenic VOCs and dry deposition (uptake by vegetation) [13].

The state of the atmosphere directly affects the emissions, transport, dispersion, and deposition of pollutants. Changes in local to regional weather affect chemical reaction rates, vertical temperature gradients, and boundary layer heights. This may affect the vertical mixing of pollutants, synoptic airflow patterns that govern pollutant transport processes, and the presence of clouds (reducing the available solar radiation), which can modify the photochemical O_3_ chain and heterogeneous reactions [7]. Further, these atmospheric responses may induce changes in natural emissions, which influence both local air quality and also global atmospheric composition [8]. In particular, higher temperatures can increase the biogenic emissions of isoprene, a volatile hydrocarbon and O_3_ precursor emitted by vegetation in significant quantities [9,14]. The indirect radiative forcing impacts from tropospheric O_3_ may be large enough for O_3_ precursors to be considered in the basket of trace gases through which policy-makers aim to combat global climate change [15]. Future O_3_ concentrations depend on not only weather and climate but also other influences such as changes in emissions of anthropogenic and biogenic precursors. The impact in regional tropospheric O_3_ concentrations driven by changes in emissions have been projected worldwide, particularly for North America, Europe, and Asia, with impacts being both positive or negative and varying by region under different climate and emission change scenarios. Some studies (e.g., References [16,17]) showed that O_3_ background concentrations have a negative trend associated with the decrease of its lifetime in regions of low NO_x_ (remote regions), leading to an increase in the O_3_ levels in the eastern and northeast parts of the United States (U.S.). The simulations of projected climate and emissions over the western U.S. showed that the effect of increasing temperature, water vapor, and biogenic VOCs emissions resulted in an increase of 1–5% in the peak concentrations of O_3_; on the other hand, the reductions of anthropogenic emissions of NO_x_, VOCs, and CO reduced O_3_ by 10–50%, 50–70%, and 8–15% respectively, in urban areas (regions with high NO_x_ concentrations).

These projections suggest that both emissions and climate change will increase the concentrations of ground-level O_3_, mainly in developed countries and Asia, but few projections are available for other less industrialized countries [18]. Determining the changes of surface O_3_ in a future climate is complex, and impacts vary regionally and are strongly influenced by changes in temperature, humidity, and hemispheric transport patterns [19]. On the other hand, many studies performing global-scale projections of O_3_ and PM_2.5_ account for climate scenarios and emission scenarios [14,19]. The downscaling scenarios of global climate models for the region of Brazil with different models considering RCP4.5 and RCP8.5 showed an increase of temperature ranging from 2 to 9 ^◦^C depending on the scenario/model, and also a decrease of the total precipitation extending the length of the dry season by 2100 [20–22]. Downscaling using the Meteorological Research Institute atmospheric general circulation model (MRI-AGCM) showed an increase in wet-season precipitation and a decrease in dry-season precipitation over most of South America, and also a large increase of consecutive dry days over the western part of the Amazon [23]. With a dryer and warmer atmosphere, a systematic increase is expected in the extreme levels of dangerous fires that can result from an increase of 2 ^◦^C by 2100 considering the RCP4.5 [24]. One difference between global model results is that in the warmer environment and with more moisture, the tropospheric ozone burden and lifetime will decrease significantly [14,19] but projections using downscaling of climate models predict the increase of the future temperature and decrease of humidity for Brazil. However, an accurate assessment of the scale (local, regional, or global) and direction of change (improvement or deterioration) of air quality is challenging [25,26]. For Brazil, some work was performed for São Paulo to study the effects of future scenarios on the weather. To the best of our knowledge, no prior studies have examined the effect on future concentrations of both O_3_ and PM_2.5_ concentration under different emission scenarios for Brazil.

This paper presents the results of a modeling framework that considers the interactions of future emissions for the RCP4.5 climate scenario to predict air quality for future decades in Brazil. Simulations using three emissions projections (current legislation emissions, mitigation, and maximum feasible reduction) for the years of 2020 and 2050 are presented (results for the years 2030 and 2040 are presented in the Supplementary Material). The main objective of this work is to analyze the sensitivity of the air quality to the changes in anthropogenic emissions for Brazil and to determine the signal and spatial patterns related to changes in O_3_ and PM_2.5_ concentrations. The results are discussed with the aim of providing scientific evidence for decision-makers and guidance for future work for the scientific community.

## 2 Methodology

The modeling system used to simulate air quality consists of the Weather Research and Forecast with Chemistry (WRF-Chem, version 4.0.2) model [27] and the updated version of the R-package EmissV [28] used to process emissions from Greenhouse Gas and Air Pollution Interactions and Synergies (GAINS) model [29]. They are described below along with WRF-Chem model evaluation protocol and observational datasets.

### 2.1 The WRF-Chem Model and Simulation

The Weather Research and Forecasting (WRF) is a multi-scale numerical prediction system consisting of a non-hydrostatic model with several physical parameterizations representing the unsolved processes by the model (sub-grid processes) [30]. It has been updated annually to incorporate state-of-the-art weather models. The WRF core integrates the mass and scalar conservation equations discretized by finite volume with fifth-order for the horizontal flux divergence (advection) in the scalar equation and third-order in the vertical flux divergence, coupled to a third-order integration scheme of Runge-Kutta. The chemical component model (Chem) is designed to simulate a variety of physical and chemical processes, such as atmospheric emission, transport, chemical reactions, aerosol formation and interactions with radiation and clouds, and wet and dry deposition. A key feature of WRF-Chem is that all modelled processes (e.g., emissions, transport, radiation, and chemistry) are fully coupled (interacting with each other) and solved simultaneously without the need of any type of interpolation [27]. Table 1 shows the WRF-Chem configurations used in this work.

The chemical mechanism used is the Carbon Bond mechanism version Z (CBMZ) [37]. It is based on the CBM-IV that includes reactive long-lived species and their intermediates. For organic species and reactions, it considers a lumped structure approach based upon similar carbon bonds, and the present version considers isoprene, SO_2_, and Dimethylsulfide chemistry. The aerosol module is the Modal Aerosol Dynamics Model for Europe (MADE) [40], in which the particle size distribution is approximated by three lognormally distributed modes (i.e., Aitken, accumulation, and coarse) in order to simulate aerosol thermodynamic equilibrium for sulfate, nitrate, ammonium, and water, and dynamic processes such as nucleation, coagulation, and condensation. This module is coupled with the secondary organic aerosol model (SORGAM) [41] for hydrophobic condensable organic compounds. The formation of new sulfate particles from homogeneous binary nucleation is simulated using a previously published approach [44]. Coagulation and condensation are simulated using a published modal approach [45]. Gas-to-particle mass transfer is simulated using the full equilibrium approach of Reference [46] for two regimes that are based on the molar ratio of total ammonium to total sulfate: ammonia-deficient regime (ratio no more than 2.0) that leads to acidic aerosol production and excess ammonium (ratio higher than 2.0), so that sulfate is completely neutralized and nitrate is produced. The input fields of the gaseous (nitrogen oxides and VOCs) and particle speciation are constructed based on previous experiments performed in São Paulo [47–49].

**Figure 1:**
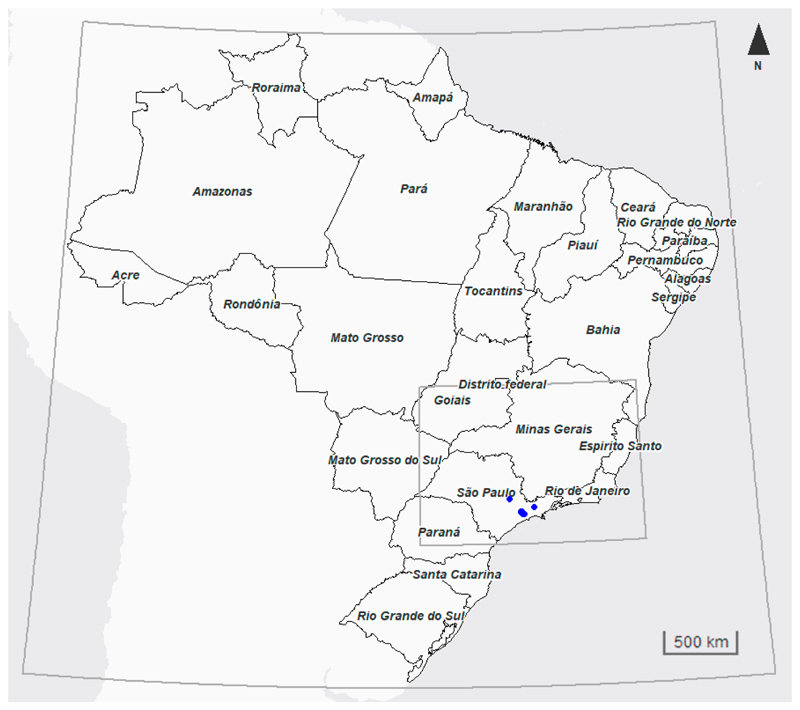
Nested domains considered in the simulation and air quality stations (blue dots).

The computational domain consists of two nested grids (Figure 1), covering the entire country of Brazil and with a nest for its Southeast region (the most highly urbanized region of the country with a large contribution to the national emissions), with 35 vertical layers. The outer domain has 126 × 126 grid-points with 36 km horizontal grid spacing and the inner domain has 157 × 121 points with 9 km horizontal grid spacing. The static data (e.g., topography, land mask, vegetation) used are provided by the United States Geological Survey (USGS) (http://www2.mmm.ucar.edu/wrf/users/download/get_sources_wps_geog.html).

Table 2 summarizes all simulations in this work along with the meteorological input, emissions used, and purpose of each simulation. As the first step towards the simulations for future emissions, a test case simulation (Run 1) is performed for the period 16 to 22 January 2019 to evaluate WRF-Chem performance by comparing the simulated results with observations. This time period is selected because it is a hot summer period with high O_3_ and PM_2.5_ and the observational data are available. For this simulation, meteorological initial and boundary conditions (ICONs and BCONs) are based on the Global Forecast System (GFS) reanalysis from National Centers for Environmental Prediction (NCEP). The chemical ICONs and BCONs are based on an idealized profile that consider clean environmental conditions, and the short-lived species are initialized to steady-state equilibrium [27]. The emissions are based on the current legislation (CLE) scenario for 2020 (CLE 2020) from the Evaluating the Climate and Air Quality Impacts of Short-Lived Pollutants (ECLIPSE) project [45,46]. Ten simulations are performed for a period of 12 days from 31 July to 10 August 2020 (the first two days are the model spin-up period, and thus not included in analysis), including one reference simulation (Run 2) and nine senstivity simuations (Runs 3–11). This time period is chosen to represent a dry period with low precipitation, no passing frontal systems, and low cloud cover (especially in the central west region) that can potentially lead to high pollutant concentrations. The meteorological conditions were particularly dry over Brazil, with the exception of the northern region: the states of Roraima and Amazonas with accumulated precipitation less than 10 mm/day on average and the states of Acre, Amapa, north of Para, and littoral of Maranhão, with accumulated precipitation more than 10 mm/day on average. No presence of frontal systems are common over the South region and Southwest region that could cause precipitation and also vertical transport of pollutants. Nine of the ten simulations are designed to examine the sensitivity of the model predictions to anthropogenic emissions of short-lived pollutants under the three scenarios from ECLIPSE (the scenarios are described in the next session). These include the emission projections under the CLE scenario for the years of 2020, 2030, 2040, and 2050 (Runs 2–5), and those under the mitigation (MIT) scenario for 2030, 2040, and 2050 (Runs 6–8), and the maximum feasible reduction (MFR) for 2030 and 2050 (Runs 9–10) (see a more detailed description on these emission scenarios in the Section 2.2 below). The sensitivity of the model predictions to anthropogenic emissions can be examined by comparison of Runs 3–10 with Run 2. While Runs 1–10 use the Guenther scheme for biogenic VOCs emissions, Run 11 uses the Model of Emissions of Gases and Aerosols from Nature (MEGAN) [50] to compare the sensitivity of the model predictions to Biogenic Volatile Organic Compounds (BVOCs) emissions. Despite a short simulation period due to limited computational resources, these simulations can help screen out the most plausible scenarios that can potentially lead to air quality benefits for future longer-term simulations when computational resources become available, and the results can provide useful insights into the responses of air quality to anthropogenic emission under these scenarios. They can also help understand the importance of BVOCs emissions in the simulation region.

The following species are included in the simulations: carbon monoxide (CO), nitrogen oxides (NO_x_), non-methane VOCs (NMVOCs), ammonia (NH_3_), particulate matter with aerodynamic diameters no larger than 2.5 and 10 µm (PM_2.5_ and PM_10_, respectively), and sulfur dioxide (SO_2_). EmissV [28] pre-processor is used to process the ECLIPSE-v5a emissions for the WRF-Chem model. The NMVOCs and PM emissions are divided into model species using data that represent conditions for Brazil [51–53]. The emissions for all the available sectors (agriculture, commercial, and residential, energy production, industrial, waste treatment, and waste burn) are combined using temporal profiles for different sectors [54], and the emissions from the transport sector are combined using profiles from mechanized traffic count at the Brazilian state of São Paulo (see Figures S1 and S2 for more details in the Supplementary Materials). EmissV has functions designed to perform spatial interpolation (to the 36 and 9 km grids) and properly transform annual averages to hourly emissions with the use of activity factors. Emissions from wildfires or biomass burn are included in ECLIPSE-v5a.

For meteorological ICONs and BCONs for the sensitivity simulations, the bias-corrected climate dataset [49] from version 1 of NCAR’s Community Earth System Model (CESM) were used, which supported the Intergovernmental Panel on Climate Change Fifth Assessment Report (IPCC AR5). The variables have been bias-corrected using the European Centre for Medium-Range Weather Forecasts (ECMWF) Interim Reanalysis (ERA-Interim) fields for 1981–2005, following the method in Reference [55]. All the sensitivity simulations were performed under the Representative Concentration Pathway (RCP) future scenario RCP4.5 [56]. RCP4.5 is a low-to-moderate emissions scenario with GHG radiative forcing reaching 4.5 W m^−2^ by 2100. It represents a trajectory that may be plausible if, for instance, GHG emissions controls were introduced to limit radiative forcing [57]. The chemical ICONs and BCONs are based on clean environmental conditions and steady-state equilibrium for the short-lived species.

The percentage change in concentrations, ΔC, were calculated by comparing the time-average of the surface concentrations under different emission scenarios or using different BVOCs emissions (i.e., sensitivity Runs 3–11) with the reference scenario (i.e., Run 2 with CLE emissions for 2020 and RCP4.5 meteorology) as follows:

(1) ΔC=100×(sensitivityrun/referencerun−1)

### 2.2 Anthropogenic Emission Scenarios

The atmospheric pollutant emissions from the RCP scenario focus on building future emission scenarios with different radiative forcing for long-lived GHG, while for air pollutants, a very similar path was assumed, strongly linked with the economic growth [31]. Consequently, all air pollutant emissions decline strongly towards 2050 in all RCP scenarios. The emission of short-lived pollutants can take a very different path depending on technology uses and economic factors, which can vary from region to region. For this reason, the emissions scenarios of the Evaluating the Climate and Air Quality Impacts of Short-Lived Pollutants (ECLIPSE) project is used (described in the next section). The emission projection and the spread (the difference between scenarios) are larger than that presented in RCP despite the fact that all scenarios follow the same energy use projection [58].

The ECLIPSE-v5a global emission dataset [59,60] describes realistic and effective short-lived climate pollutants mitigation scenarios for the recent past and future. These emissions were created with the Greenhouse gas–Air pollution Interactions and Synergies (GAINS) model [31]. GAIMS provides emissions of shorter-lived species as well as the long-lived GHGs in a consistent framework that considers different national and regional strategies to respond to global and long-term climate objectives (expressed in terms of GHGs emissions), while maximizing the local and short- to medium-term environmental benefits of air pollution reduction.

The GAINS model holds essential information about key sources of emissions, environmental policies, and mitigation opportunities for about 170 country-regions and considers more than 2000 technologies to control air pollutant emissions (developed with the help of air quality modeling results for different areas). The model relies on exogenous projections of energy use, industrial production, and agricultural activity for which it distinguishes all key emission sources and control measures. This allows a full capture of interactions between pollutants for each individual emission control measure. In such a way, the traditional cost curve approach of the RAINS model [61] is replaced by a technology-driven problem formulation [62]. The GAINS emissions are calculated from three input parameters: annual activity levels in a given sector based on the information from international statistics, shares of abatement technologies applied to fuel consumption of the activity, and emission factors by sectors-fuel-technology-combination [59].

The global emissions from the ECLIPSE (version v5a) are available as yearly means from 1990 to 2050 with a spatial grid resolution of 0.5^◦^ × 0.5^◦^ longitude-latitude and divided into seven activity sectors: transport covering road and non-road transportation sources including tire and brake wear and road abrasion [63], energy generation from power plants, energy production/conversion, and fossil fuel distribution [64,65], industrial combustion and process [66], residential and commercial combustion sources [67], agriculture and agricultural waste burn that covers livestock and arable land operations, such as plowing and harvesting and open burn of agricultural residues [68], solvent use [64], and waste treatment including waste disposal and trash burning [69].

The global anthropogenic emissions were considered for three developed scenarios for short-lived pollutants: the current legislation (CLE) scenario, mitigation (MIT), and the maximum feasible reduction (MFR). The CLE scenario has higher emissions than the MIT scenario for most pollutants, followed by the MFR that represents the drastic reduction for all pollutants [59]. The reference scenario is CLE, where the existing legislation (frozen by the year 2015) is implemented but there are no assumptions made as to how such legislation can develop further in the coming decades for various regional (Europe, Asia) and global projects. Primary sources of data for the development of activities originate from international modelling studies, while the technological parameterization and implementation rates consider peer-reviewed data on emission performance of various technologies and emission limit values and their implementation rates as defined in national laws. However, the CLE scenario still might be optimistic as it does not assume any failure or further delays in enforcement of pre-2015 laws. The MIT scenario includes many actions with the goal of improved air quality and reduced climate impacts such as technological improvements on key sectors as a measure to reduce emissions of both short-lived pollutants and GHGs. The MFR is a scenario developed for 2030 and 2050, in which the best available technology is applied to all source sectors. This scenario assumes the unconditional implementation of technologies with the lowest emission factors but no introduction of non-technical measures that would improve resource efficiency and lead to a significant change of energy balance. The scenario ignores possible constraints, either technical, institutional, or cultural, that would still be in place by 2030 or 2050 in some regions.

**Figure 2:**
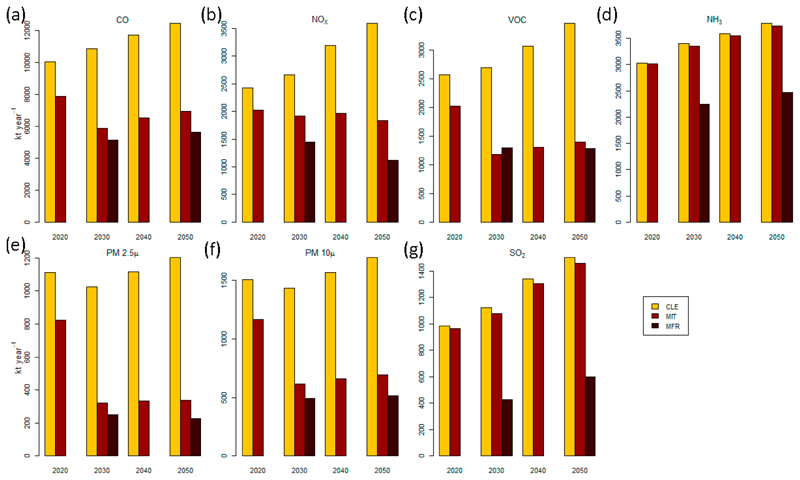
Annual emissions of (**a**) CO, (**b**) NO_x_, (**c**) NMVOCs, (**d**) NH_3_, (**e**) PM_2.5_, (**f**) PM_10_, and (**g**) SO_2_ for Brazil.

Figure 2 shows the total projected emissions of ECLIPSE scenarios over Brazil for these species for 2020–2050. The CLE scenario (in yellow) shows an increase of the total emissions due to the lack of new measures to reduce emissions and the increase of activity, the MIT scenario (in red) shows a significant reduction of the total emissions, and the MFR scenario (in brown) shows a more drastic reduction (comparing with the MIT totals). The CLE and MIT scenarios are available for the years from 2020 to 2050, while the MRF emissions are available only for the years 2030 and 2050.

### 2.3 Evaluation Protocols and Observational Datasets Used for Evaluation

Traditional statistical indexes are used in the model evaluation including Pearson correlation coefficient (r), the factor of two (FA2), root mean square error (RMSE), mean bias (MB), and normalized mean bias (NMB) [70]. The criteria for a good model performance include: r exceeding 0.75 or 0.70 for a good performance related to O_3_ or PM_2.5_ respectively, or larger than 0.5 or 0.4 for an acceptable performance related to O_3_ or PM_2.5_ respectively, and NMB less than 15% or 30% for O_3_ or PM_2.5_ respectively, for an acceptable performance, and 5% or 10% for good performance [71].

The model evaluation in this work focuses on the 9 km domain. The results from the 36 km domain are, therefore, only compared against the observations obtained for locations within the fine-grid domain. The model performance of O_3_ is evaluated using hourly data from 6 automatic air-quality monitoring stations located at urban sites (Americana, Maua, N. Senhora do Ó, Pinheiros, Santana, and Sao Caetano do Sul), provided by the São Paulo State Environmental Agency (CETESB) network [72]. The model performance of PM_2.5_ is evaluated using daily observations that are calculated based on hourly data from four stations at urban sites (Mocca, Pinheiros, Santana, and São José dos Campos) from CETESB. The location of the CETESB stations are marked in Figure 1 as blue dots. The two first simulated days are considered model spin-up and thus, are not included in the evaluation.

## 3 Results

### 3.1 Model Performance Evaluation

As the first step towards the simulations for future emissions, a test case simulation was performed to evaluate its performance by comparing the simulated results with observations. The simulation covered a hot summer period (from 16 to 22 January 2019), characterized by high temperatures, low wind speed, and cloudless conditions over the region where air quality data was available. These factors favor O_3_ production and decrease the impact of transport and dispersion processes. This period was chosen because the air quality data was available, and it covers a recent period compatible with the emissions of CLE for 2020 that represent the current emissions. The meteorological data for the initial and boundary conditions of the test case are from the GFS model with 0.25^◦^ grid resolution and the emissions are from the CLE scenario for 2020.

**Figure 3:**
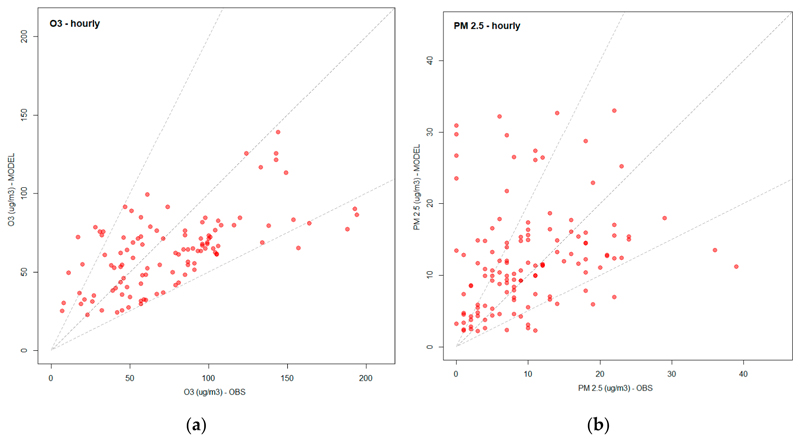
Scatter plot of hourly observed versus simulated over the 9 km domain (**a**) O_3_ (µg/m^3^) and (**b**) PM_2.5_ (µg/m^3^).

Figure 3a shows the scatter plot between the hourly observed and simulated O_3_ concentrations from the 9 km domain. The central gray line represents the 1:1 ratio, and the upper and lower lines represent twice and half of the observed O_3_ value, respectively. Most of the points are concentrated around the central line, indicating that the simulated value is similar to the observed value. Figure 3b shows the scatter plot of the observed and simulated PM_2.5_ concentrations, the simulated values are more spread than the O_3_ case and many points are above the gray line of twice the observed values, indicating moderate-to-large overpredictions.

In order to quantify the model errors, the five statistical indexes are calculated using hourly values of O_3_ concentration and 24 h average for PM_2.5_ concentration as described in Reference [69] using the 9 km domain results. The Pearson correlation index (r) is between 0.57 and 0.96 for O_3_ (which is considered to indicate reasonable model performance) and −0.17 and 0.54 for PM_2.5_ (representing poor model performance). The FA2 ranges from 0.69 to 1.0 for O_3_ and is 1.0 for PM_2.5_ for all stations. The RMSE is 28.18 µg/m^3^ for O_3_ and 3.8 µg/m^3^ for PM_2.5_, respectively. The MB for O_3_ is 0.86 µg/m^3^ (2.13 µg/m^3^ for PM_2.5_), and NMB for O_3_ is −4.39% (17.57% for PM_2.5_), which is good for O_3_ and reasonable for PM_2.5_ [70,71]. The model underpredicted the concentration of O_3_ by ~5% and overpredicted PM_2.5_ by ~17%. Such differences between observed and estimated values may be caused by the period of the year that has less emissions due to periods of scholarly vacation in Brazil. This period has reduced traffic activity and consequently, vehicular emissions are reduced. Emissions from other sectors are also affected, making it difficult to precisely determine emissions. These results show that large uncertainties are related to the annual emissions from ECLIPSEv5a [54,55] used in this work, in particular, the temporal profiles (for example, stations that have a negative correlation to PM_2.5_) for each sector activity and the spatial patterns (the model shows a very different performance at different sites).

The following sections present the sensitivity simulation results considering different anthropogenic emissions scenarios (i.e., CLE, MIT, and MFR) for the year 2050 and by comparing their predictions of mean surface concentrations of O_3_ and PM_2.5_ with those from the reference scenario using CLE emissions for 2020 (i.e., Run 1), to avoid the rainy period (i.e., January), a different period was chosen (31 July to 10 August) to perform the Run 2 to Run 11 considering the RCP4.5 scenario.

### 3.2 Current Legislation Scenario

**Figure 4:**
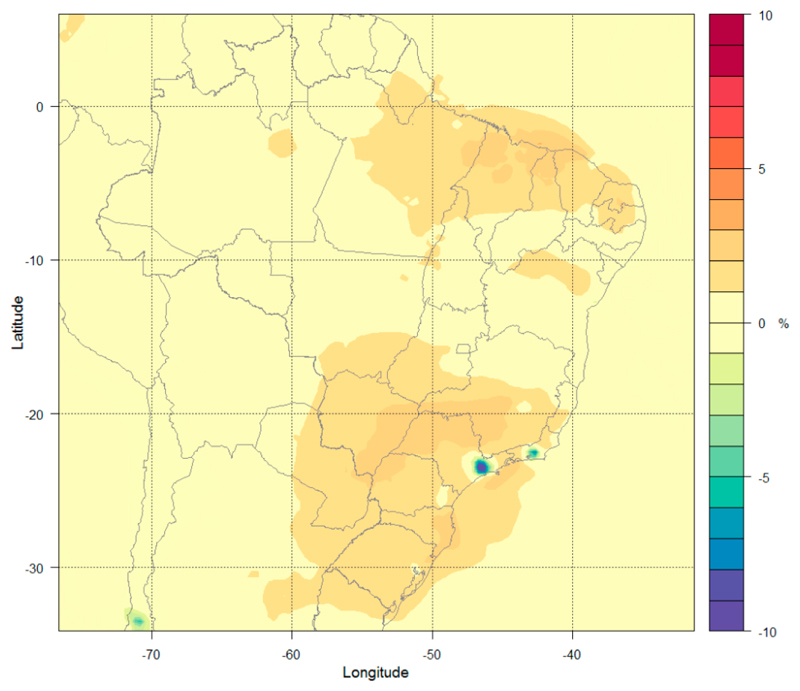
Percentage variation in surface O_3_ concentrations (%) under the CLE-2050 emission scenario.

The simulations for the CLE are performed using emissions from all sectors for the years 2020, 2030, 2040, and 2050, along with meteorological conditions from the CESM Bias-Corrected dataset for the RCP4.5 scenario [49] for the year 2020 processed by the WPS (WRF pre-processing system). The meteorological conditions for the simulated period were particularly dry without precipitation, with the exception of some states in the northern region. The states of Roraima and Amazonas had regions that presented precipitation up to 10 mm/day and the states of Amapá, the north of the states of Acre, Pará, and the littoral of Maranhão, had accumulated precipitation that reach 5 mm/day over the simulated period. There is no presence of frontal systems on the simulated period. Figure 4 shows the changes in O_3_ concentration at the surface level using CLE 2050 (see a similar plot for 2030, 2040 and 2050 in the Figures S3 and S4 in Supplementary Materials) emission projections (Run 5) relative to the reference scenario (Run 2) based on the 36 km domain results (see a similar plot for the 9 km in Figure S5 in the Supplementary Materials).

The variation in surface O_3_ concentrations from 2020 to 2050 can be summarized into two main groups. The first group in which the concentrations increase by 1–3% is located at two large regions in the center and south of Brazil and another one in the Southeast region. A second group has a decrease up to 10% in O_3_ concentrations and is located at two small regions that are the Metropolitan Area of São Paulo (MASP) and the Metropolitan Area of Rio de Janeiro (MARJ). This difference is caused mainly by the increase of NO_x_ emissions in different NO_x_/VOCs regimes. In urban areas, the reduction of NO_x_ leads to the reduction of O_3_, while the O_3_ increase occurs in other regions (with lower NO_x_/VOC ratios).

**Figure 5:**
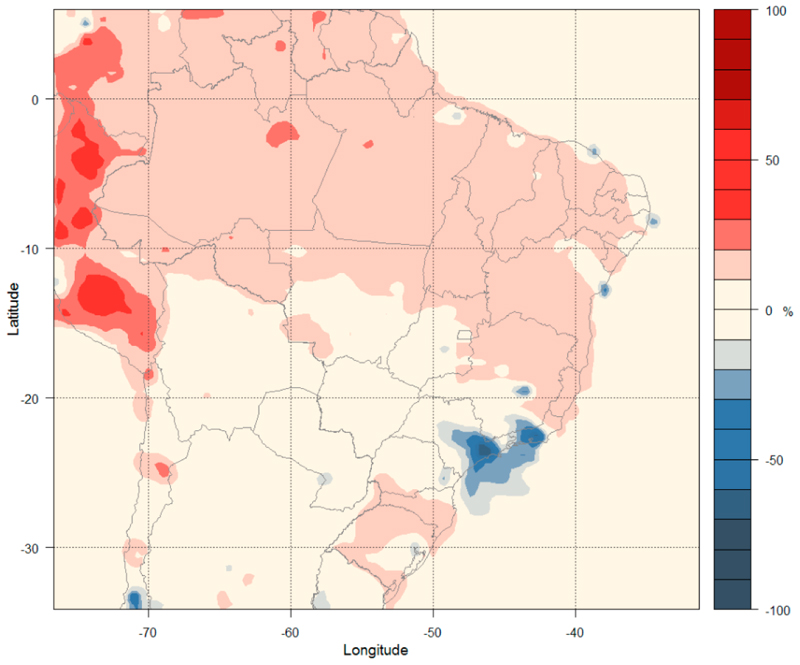
Percentage variation in surface PM_2.5_ concentrations (%) under the CLE-2050 emission scenario.

This scenario considers the increase of PM_2.5_ emissions in all domains, with the exception of the state capitals and the metropolitan regions of São Paulo and Rio de Janeiro, metropolitan regions with a very small decrease of PM_2.5_ on air quality. Figure 5 shows the percentage variation in PM_2.5_ concentration at the surface level from the model simulations at 36 km (a similar plot for 9 km is presented in Figure S6 in the Supplementary Materials). The concentrations increase from 10% to 15% for almost all of Brazil (delimited by the red area of Figure 5). The regions around the MASP and MARJ show a decrease of up to 27% in PM_2.5_ concentration. The same behavior is found locally, close to other big urban centers.

Table 3 shows the mean and the range of variation (across all grid cells at 9 or 36 km in each state) for O_3_ and PM_2.5_ concentrations by comparing Run 5 to Run 2 for each Brazilian state. The values for the states of Espirito Santo, Minas Gerais, Rio de Janeiro, and São Paulo are extracted from the 9 km domain, while values for the remaining states are extracted from the 36 km domain. An increase of 1–2% on average for O_3_ is estimated, with the exception of the Rio de Janeiro state, and an increase of 10% for PM_2.5_ on average is estimated, with the exception of MASP and MARJ, where a decrease of 27% and 15% respectively, occurs.

The above changes in concentration predictions are attributed to changes in the emissions that increase due to expansion of sector activities. The metropolitan areas have some policies to reduce emissions, which results in improved air quality in each metropolitan area. The changes over MASP and MARJ are important, showing not only the higher reduction in concentrations but also a large affected region far away from the metropolitan area for PM_2.5_.

### 3.3 Mitigation Scenario

**Figure 6:**
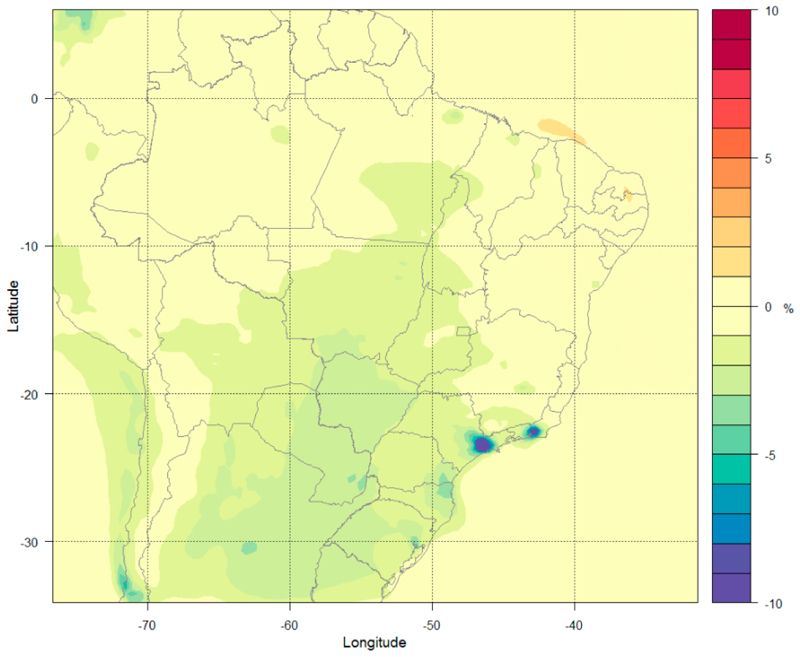
Percentage variation of surface O_3_ concentrations (%) under the MIT-2050 emission scenario.

The MIT scenario incorporates beneficial measures taken for air quality control and the reduced emissions from key sectors of activity due to technological improvements. These simulations are performed using emissions for all sectors for the years 2030, 2040, and 2050 along with meteorological data of the RCP4.5 scenario for 2020. Figure 6 shows the percentage variation of O_3_ concentration at the surface level calculated using Equation (1) between Run 8 and Run 2 for the 36 km domain (see a similar plot for the 9 km in Figure S5 in the Supplementary Materials). The main feature in projected O_3_ changes is a reduction in concentrations by 1–2% over a large area from the Midwest and Para state regions to the south of Brazil. This is the result of the reduction of NO_x_ combined with a strong reduction of NMVOCs in all regions. The plot for the 9 km is presented in Figure S5 in the Supplementary Materials.

**Figure 7:**
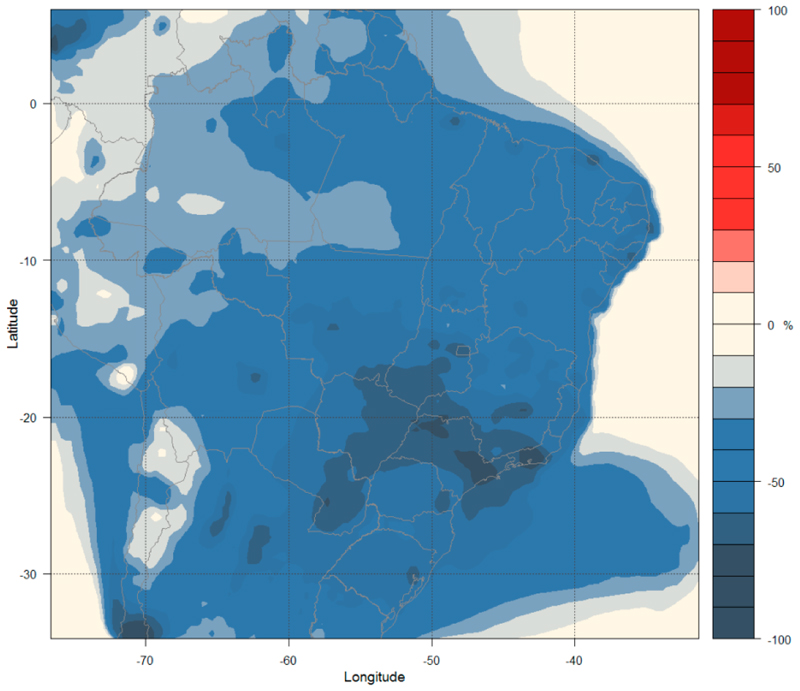
Percentage variation in surface PM_2.5_ concentrations (%) under the MIT-2050 emission scenario.

This scenario considers a strong decrease of PM_2.5_ emissions. Figure 7 shows the changes in PM_2.5_ concentrations at the surface level for the 36 km domain (similar plot for the 9 km is presented in Figure S6 in the Supplementary Materials). The MIT scenario shows a decrease by 30–60% of PM_2.5_ concentrations. The MASP and MARJ show the largest area of influence with a reduction of up to 80% in concentrations.

Table 4 shows the mean and the range of the changes in O_3_ and PM_2.5_ concentrations by comparing Run 8 and Run 2 for each Brazilian state. On average, there is a decrease of 1–3% in O_3_ concentrations and a decrease of 30–60% in PM_2.5_ concentrations.

The central and south parts of Brazil have substantial improvement in air quality in relation to O_3_, but the Southeast and Midwest metropolitan areas showed a stronger improvement in air quality related to reduction of ozone concentrations. The results showed clear improvement in air quality in relation to PM_2.5_ concentrations for all of Brazil from measures adopted on the MIT scenario and the Southeast and Midwest regions have a greater impact in PM_2.5_ concentrations.

### 3.4 Maximum Feasible Reduction Scenario

**Figure 8:**
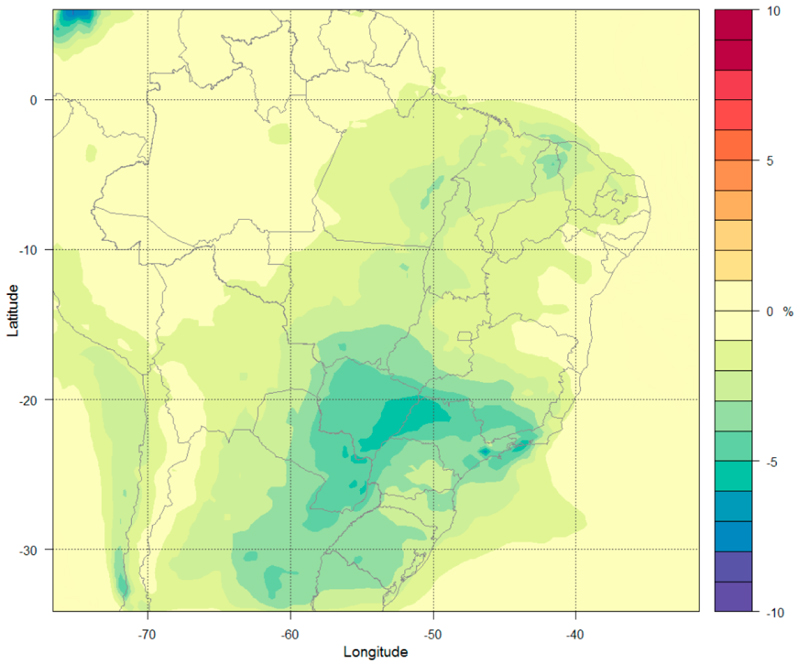
Percentage variation in surface O_3_ concentrations (%) under the MFR-2050 emission scenario.

The Maximum Technically Feasible Reduction (MFR) scenario assumes the implementation of the best available measures without political or economic constraints but considering technical limitations (applicability’s) that might vary regionally. The simulations are performed with emission projections for 2030 and 2050 only (Runs 9–10 in Table 2). Figure 8 shows the changes in O_3_ concentration at the surface between Run 10 and Run 2 for the 36 km domain (a similar plot for the 9 km is presented in Figure S5 in the Supplementary Materials). The O_3_ concentrations decrease with the highest reduction of 4–5% over the states of São Paulo and Mato Grosso. The South region (Rio Grande do Sul, Santa Catarina, and Parana) has a reduction of 3%, the central region (some states from West, North, and Northeast) shows a decrease of 2%, and other regions have 1% decrease. The decrease of O_3_ is the result of the reductions in its major precursors such as NO_x_, CO, NMVOCs, and methane.

**Figure 9:**
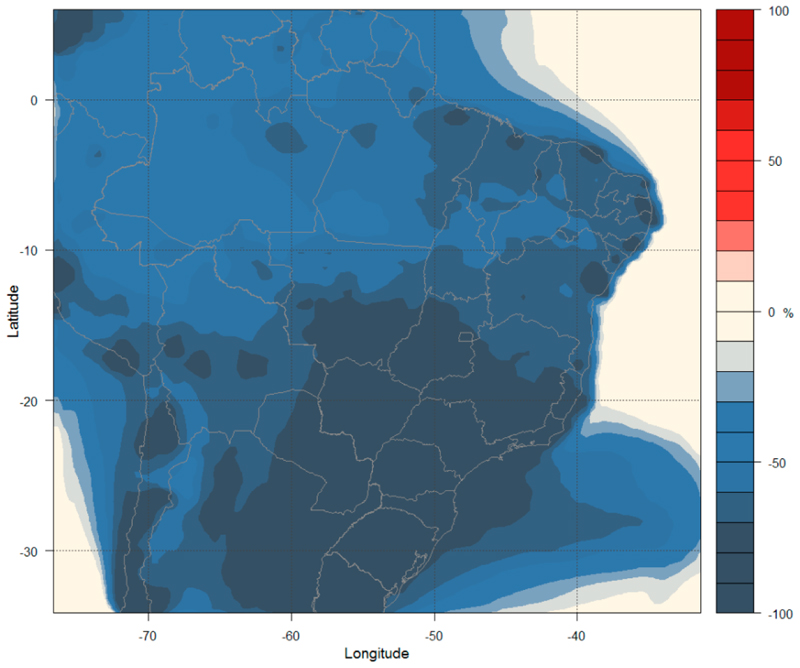
Percent variation in surface PM_2.5_ concentrations (%) under the MFR-2050 emission scenario.

Figure 9 shows the changes in PM_2.5_ concentrations at the surface level, which decrease over the entire study area (a similar plot for the 9 km is presented in Figure S6 in the Supplementary Materials). The Midwest, Southeast, and South regions show a reduction of 75% on average, while the North region shows a reduction of 50% and the Northeast region shows a reduction of 65%.

This scenario considers a drastic decrease of PM_2.5_ emissions. Table 5 shows the mean and range of changes in O_3_ and PM_2.5_ concentrations by comparing Run 10 and Run 2 for each Brazilian state.

O_3_ concentrations are projected to decrease by 0.23–4.6% and PM_2.5_ concentrations are projected to decrease by 46.77–82.77%.

The MFR scenario represents a significant air quality improvement when compared with other scenarios for both O_3_ and PM_2.5_ concentrations. The reductions in concentrations of PM_2.5_ and O_3_ occur in both the less polluted regions (with small reductions) and the most polluted areas (with greater reductions), showing that the emissions projection under this scenario is very effective for air quality improvement.

## 4 Conclusions

In this work, the WRF-Chem model was applied to Brazil to evaluate the model performance against observations and to study the sensitivity of air quality predictions to changes in emissions. A simulation during a hot summer episode in 2019 was used to evaluate model performance, which showed reasonable agreement with observed data for surface concentrations of O_3_ and PM_2.5_. As the first step towards long-term model simulations, the present work examined the changes in O_3_ and PM_2.5_ concentrations due to different emission projections under the RCP4.5 climate scenario using short-period simulations. Each of these scenarios was briefly discussed in terms of its basic characteristics, and simulations of air quality were performed using projected emissions in 2020 and future years under three emission scenarios. The absolute and percentage concentration changes were calculated by comparing the sensitivity simulations with the reference scenario simulation.

Future air quality depends partially on the concentrations of pollutants at the global and regional scale, climate future conditions, and emissions. Uncertainties are present in emission inventories, including the extent of future changes in emissions, related to the assumptions of population growth, economic development, technological development and integration, regulatory actions, and energy use. Additionally, many uncertainties are associated with biogenic emissions and representation of chemical processes of O_3_ and PM_2.5_ from biogenic VOCs in the models. These are the main sources of uncertainty of the input data, as well as pollutant sources that are not considered in the emissions.

The comparison among different emission scenarios (without including climate change) shows that the MFR is the scenario with the greatest improvement of air quality, presenting a mean concentration reduction of 3% in O_3_ and 75% in PM_2.5_, with reductions covering a large area across Brazil. The most affected states were São Paulo and Mato Grosso do Sul, with 4.5% O_3_ reduction. The CLE emission scenario presented the worst air quality related to emission changes, presenting a mean concentration increase of 1% in O_3_ and 11% in PM_2.5_. Some metropolitan regions showed reduced concentrations but occurred very locally.

The PM_2.5_ and O_3_ concentration responses to changes of emissions were non-linear and non-homogeneous over the Brazilian states. The patterns of O_3_ changes projected by the emission scenarios were related to local emissions of big metropolitan centers, mainly in São Paulo and Rio de Janeiro metropolitan areas, and they affect an influence zone next to these centers. The PM_2.5_ concentrations were also related to emissions in big metropolitan centers, showing a strong pattern of decreased concentrations in the South-Central and Southeastern regions.

Due to the importance in the atmospheric process of formation of O_3_ and PM_2.5_ and the largest uncertainties in future BVOCs emissions (due to changes in both climate and vegetation), a sensitivity simulation was performed using another online biogenic emission scheme available in the WRF-Chem model, the MEGAN 2 [50], and compared with the reference scenario using the Guenther scheme [42,43] (Run 2). Both simulations were performed using anthropogenic emissions from CLE 2020 and meteorological input form RCP4.5. These sensitivity tests showed that the prediction of O_3_ and PM_2.5_ concentrations has a strong dependence on biogenic emissions for the Amazon region but a small dependence in other regions. The range of the changes in PM_2.5_ concentrations was very small (on the order of 0.01 µg/m^3^), although such a small change occurred over all the study regions, with the exception of the Northeast region. A reduction of PM_2.5_ up to 30% was found in the states of Acre, Amazonas, and Roraima. The difference in O_3_ ranged from −17 µg/m^3^ (in the north of the states of Amazon and Roraima) to 1.2 µg/m^3^ (over the ocean areas next to the coast), while a reduction of 25% was estimated for the north region reaching 60% in the north of the state of Amazonas. This reduction of O_3_ and PM_2.5_ in remote regions is due to differences in the estimated emissions of biogenic VOCs between MEGAN 2 and the Guenther scheme; thus, the biogenic emissions are an additional source of uncertainty for the projection of O_3_ and PM_2.5_ for this region.

There are several limitations for this work. First, this work used annual emissions from a global emission inventory. The results indicate that more detailed emissions are necessary for a complete analysis of the future O_3_ and PM_2.5_ changes over Brazil, due to the high heterogeneity of spatial distributions of economic and natural resources across different regions of the country. Important aspects that must be considered are the emission factors and activity profiles that vary over time and space. Second, realistic scenarios considering higher emissions than CLE are also needed to investigate future delays and failures on emission control policies. Scenarios on how the urban environment and land use will evolve in the next decades could also be an important inclusion for future work, especially for studies focusing on local air quality. Third, the biogenic emissions of the Guenther scheme used in most simulations warrants updating. Future studies that focus on biogenic emissions should adopt a more recent biogenic emission module such as the MEGAN 2.0 (or newer versions when they become available) and should also account for changes of vegetation, such as the reduction of biodiversity and extension of forests and vegetated areas. Finally, simulations of longer time periods are needed in the future when computational resources become available to address the degree to which climate and emissions changes together will affect future air quality, in a more realistic way, considering the seasonal patterns, interannual variabilities, and different weather conditions in conjunction with emission changes.

## Supplementary Material

Supplementary material

## Figures and Tables

**Table 1:** Weather Research and Forecast with Chemistry (WRF-Chem) options considered in the simulations of air quality ^a^.

Parameterization	Option
Radiation	RRTMG scheme (longwave and shortwave) [31]
Surface layer	Revised MM5 Monin-Obukhov scheme [32]
Land surface	Unified Noah land-surface model [33]
Boundary layer	YSU scheme [34]
Cumulus clouds	Grell 3D Ensemble scheme [35]
Cloud microphysics	Morrison 2-moments [36]
Gas-phase chemistry	CBMZ [37]
Photolysis	Fast-J photolysis [38,39]
Aerosol/microphysics model	MADE [40]
Secondary organic aerosol	SORGAM [41]
Biogenic emissions	Guenther scheme [42,43]

a RRTMG: Rapid Radiative Transference Model; MM5: Fifth-Generation Penn State/ National Center for Atmospheric Research (NCAR) Mesoscale Model; NOAH LSM: NOAH: N—National Centers for Environmental Prediction, O—Oregon State University, A—Air Force, H—Hydrologic Research Lab, now Office of Hydrologic Dev, LSM: Land Surface Model,; YSU: Yonsei University Scheme; CBMZ: Carbon Bond mechanism version Z; MADE: modal aerosol dynamics model for Europe; SORGAM: secondary organic aerosol model.

**Table 2:** List of simulated cases.

Run Index	Meteorology	Anthropogenic Emissions	BVOCs Emissions	Purpose
1	GFS 2019	CLE 2020	Guenther	Evaluation of the model
2	RCP4.5 2020	CLE 2020	Guenther	Reference scenario
3	RCP4.5 2020	CLE 2030	Guenther	To study the sensitivity to changes in anthropogenic emissions
4		CLE 2040	Guenther	
5		CLE 2050	Guenther	
6		MIT 2030	Guenther	
7		MIT 2040	Guenther	
8		MIT 2050	Guenther	
9		MFR 2030	Guenther	
10		MFR 2050	Guenther	
11	RCP4.5 2020	CLE 2020	MEGAN 2	To study sensitivity to biogenic emissions

**Table 3:** Changes of surface O_3_ and PM_2.5_ concentrations (µg/m^3^) due to emission changes between CLE-2020 and CLE-2050, by state.

State *	O_3_ Mean (%)	O_3_ Range(%)	PM_2.5_ Mean (%)	PM_2.5_ Range (%)
Acre	0.22	[0.14, 0.37]	14	[10, 26]
Alagoas	0.22	[−0.06, 0.63]	8	[0,14]
Amapá	1.00	[0.64,1.40]	13	[8, 20]
Bahia	0.67	[−1.00,10.60]	13	[−27,19]
Ceará	1.50	[0.22, 2.70]	12	[−22,18]
Distrito federal	0.54	[0.42, 0.68]	0	[−7, 5]
Espirito Santo	0.80	[0.22, 1.50]	16	[10, 19]
Goiás	1.10	[0.32, 2.00]	7.2	[−17,18]
Maranhão	1.60	[0.87,2.80]	15	[−5,19]
Mato Grosso	0.76	[0.31, 1.70]	10	[2,15]
Mato Grosso do Sul	1.80	[1.10, 2.60]	3	[−3,9]
Minas Gerais	1.20	[−0.00, 2.70]	9	[−31,19]
Pará	1.00	[−0.07, 2.30]	15	[−17, 20]
Paraíba	1.20	[0.27, 2.00]	10	[−4,16]
Pernambuco	0.71	[−0.17,1.30]	11	[−12,17]
Piauí	1.20	[0.65, 2.90]	14	[5,18]
Rio de Janeiro	0.20	[−7.50, 2.00]	−17	[−60,15]
Rio Grande do Norte	1.40	[−0.07, 2.10]	10	[−3,17]
Rio Grande do Sul	1.50	[−0.09, 2.10]	9	[−19,14]
Roraima	0.33	[0.12,0.61]	15	[12, 22]
Santa Catarina	1.70	[1.20, 2.30]	11	[1,14]
São Paulo	1.40	[−11.00,2.80]	−13	[−65,4]
Sergipe	0.23	[−0.10, 0.68]	12	[9,15]
Tocantins	0.92	[0.75, 1.30]	13	[10, 19]
Paraná	1.60	[0.09, 2.30]	4	[−22,12]
Rondônia	0.31	[0.23, 0.44]	14	[9, 20]
Amazonas	0.34	[0.08,1.70]	14	[10, 25]

* Values extracted from the 36 km domain, with the exception of the states of Espírito Santo, Minas Gerais, Rio de Janeiro, and São Paulo, which are extracted from the 9 km domain.

**Table 4:** Changes in surface O_3_ and PM_2.5_ due to emission changes between CLE-2020 and MIT-2050 by state.

State *	O_3_ Mean (%)	O_3_ Range(%)	PM_2.5_ Mean (%)	PM_2.5_ Range (%)
Acre	−0.41	[−0.78, −0.29]	−29	[−42, −23]
Alagoas	−0.016	[−0.19, 0.22]	−54	[−62, −48]
Amapá	−0.24	[−0.55, −0.02]	−35	[−47, −25]
Bahia	−0.10	[−1.30,0.61]	−44	[−57, −35]
Ceará	−0.053	[−1.20,1.10]	−46	[−66, −41]
Distrito federal	−1.20	[−1.20, −1.10]	−63	[−66, −61]
Espirito Santo	−0.24	[−0.61, 0.03]	−52	[−58, −48]
Goiás	−0.24	[−0.61, 0.03]	−52.	[−58, −48]
Maranhão	−0.67	[−2.20, 0.37]	−43	[−60, −32]
Mato Grosso	−1.40	[−2.70, −0.44]	−45	[−64, −22]
Mato Grosso do Sul	−2.20	[−2.90, −1.80]	−60	[−70, −50]
Minas Gerais	−0.77	[−2.50, −0.04]	−54	[−71, −29]
Pará	−0.77	[−2.50, −0.04]	−54	[−71, −29]
Paraíba	0.38	[−0.08,1.20]	−49	[−57, −45]
Pernambuco	0.039	[−0.27, 0.46]	−47	[−60, −39]
Piauí	−0.37	[−1.50,0.24]	−40	[−54, −34]
Rio de Janeiro	−2.40	[−11.00, −0.23]	−67	[−81, −56]
Rio Grande do Norte	0.53	[−0.11,1.10]	−49	[−56, −45]
Rio Grande do Sul	−2.4	[−11.00, −0.23]	−67	[−81, −56]
Roraima	−0.21	[−0.44, −0.08]	−30	[−45, −19]
Santa Catarina	−2.10	[−3.07, −1.50]	−51	[−57, −48]
São Paulo	−2.30	[−15.00, −0.76]	−67	[−82, −55]
Sergipe	0.017	[−0.18, 0.30]	−51	[−54, −42]
Tocantins	−0.95	[−1.50, −0.45]	−41	[−51, −32]
Paraná	−2.3	[−15.00, −0.76]	−67	[−82, −55]
Rondônia	−0.64	[−1.20, −0.31]	−33	[−45, −23]
Amazonas	−0.30	[−1.50, −0.06]	−26	[−53, −14]

* Values extracted from the 36 km domain, with the exception of the states of Espírito Santo, Minas Gerais, Rio de Janeiro, and São Paulo, which are extracted from the 9 km domain.

**Table 5:** Changes in surface O_3_ and PM_2.5_ due to emission changes between CLE-2020 and MFR-2050 by state.

State *	O_3_ Mean (%)	O_3_ Range(%)	PM_2.5_ Mean (%)	PM_2.5_ Range (%)
Acre	−0.46	[−0.80, −0.34]	−46	[−58, −39]
Alagoas	−0.29	[−0.73, 0.04]	−68	[−75, −63]
Amapá	−0.90	[−1.30, −0.66]	−53	[−65, −39]
Bahia	−0.43	[−1.40, −0.09]	−46	[−69, −34]
Ceará	−0.95	[−1.60,0.81]	−64	[−83, −51]
Distrito federal	−2.00	[−3.90, −1.00]	−64	[−80, −59]
Espirito Santo	−1.60	[−1.80, −1.50]	−76	[−78, −74]
Goiás	−1.30	[−2.60, −0.41]	−71	[−74, −67]
Maranhão	−2.50	[−4.40, −1.30]	−75	[−82, −61]
Mato Grosso	−2.10	[−2.90, −1.20]	−63	[−76, −54]
Mato Grosso do Sul	−2.20	[−4.10, −0.65]	−64	[−81, −43]
Minas Gerais	−4.60	[−5.80, −3.40]	−79	[−84, −72]
Pará	−2.40	[−5.10, −0.41]	−73	[−84, −61]
Paraíba	−1.50	[−3.20, −0.46]	−55	[−80, −46]
Pernambuco	−1.20	[−1.80, −0.20]	−66	[−73, −61]
Piauí	−3.40	[−5.00, −2.00]	−76	[−82, −74]
Rio de Janeiro	−0.88	[−1.40,0.09]	−63	[−76, −56]
Rio Grande do Norte	−1.80	[−4.10, −1.00]	−60	[−72, −56]
Rio Grande do Sul	−3.10	[−5.70, −1.20]	−80	[−90, −72]
Roraima	−1.50	[−2.10, 0.04]	−65	[−73, −61]
Santa Catarina	−3.20	[−4.10, −1.80]	−75	[−82, −67]
São Paulo	−0.81	[−1.60, −0.45]	−53	[−63, −42]
Sergipe	−0.34	[−0.64, −0.14]	−48	[−61, −35]
Tocantins	−2.80	[−3.40, −2.30]	−74	[−76, −73]
Paraná	−4.30	[−7.00, −2.60]	−82	[−91, −75]
Rondônia	−0.26	[−0.66, 0.08]	−67	[−72, −59]
Amazonas	−2.00	[−2.80, −1.60]	−61	[−69, −54]

* Values extracted from the 36 km domain, with the exception of the states of Espírito Santo, Minas Gerais, Rio de Janeiro, and São Paulo, that are extracted from the 9 km domain.
