# Supplementary material for: Short-Term Responses of Air Quality to Changes in Emissions under the Representative Concentration Pathway 4.5 Scenario over Brazil
Source: Atmosphere (Basel). Author manuscript; Available in PMC 2024 May 27. (PMC7615996; doi:10.3390/atmos11080799)
Supplement: Supplementary material [file EMS196214-supplement-Supplementary_material.pdf]

atmosphere

Supplementary Material

Short-Term Responses of Air Quality to Changes in Emissions under the Representative Concentration Pathway 4.5 Scenario over Brazil

Daniel Schuch [1,*], Maria de Fatima Andrade [2], Yang Zhang [1,*], Edmilson Dias de Freitas [2] and Michelle L. Bell [3]

[1] Department of Civil and Environmental Engineering, Northeastern University, 02115, Boston, MA, USA
[2] Instituto de Astronomia, Geofísica e Ciências Atmosféricas, Universidade de São Paulo, 05508-090 São Paulo, SP, Brazil; maria.andrade@iag.usp.br (M.d.F.A.); edmilson.freitas@iag.usp.br (E.D.d.F.)
[3] School of Forestry & Environmental Studies, Yale University, New Haven, CT 06511, USA; michelle.bell@yale.edu
* Correspondence: d.schuch@northeasthern.edu (D.S.); ya.zhang@northeastern.edu (Y.Z.)

Received: 6 July 2020; Accepted: 27 July 2020; Published: date

1. Temporal Profiles and Speciation of the Emission

The ECLIPSE-v5a total emissions were divided into seven activity sectors. To combine the data from these different sectors, we used daily activity profiles by sectors from [54] showed in Figure S1a. For the transportation sector, we used alternative profiles from volumetric mechanized traffic count at Anhanguera-Castello Branco highways performed in October 2018 at São Paulo City-Brazil, which accounts for the daily profile and also the weekday regime. This is shown in Figures S1b,c.

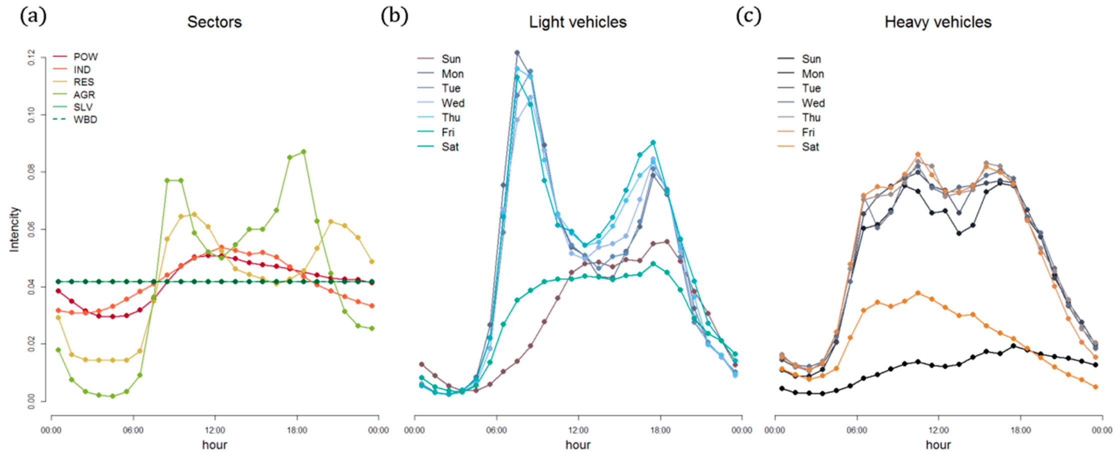

Figure S1. Activity profiles for (**a**) emission sectors, (**b**) light vehicles and (**c**) heavy vehicles.

The lines in Figure S1a represent the profiles for six activity sectors: POW represents the energy generation activity, IND represents the industrial activity, RES represents the domestic emission activity, AGR represents the emission profile from agricultural, and SLV and WBD represent constant activity for solvent use and waste burn, respectively. In Figure S1b,c the lines represent the activity for different weekdays for the transportation sector for light and heavy-duty vehicles.

Figure S2 shows the mass fraction of the ECLIPSE-v5a emissions mapped into the model species. The nitrogen oxides emissions (NO_X) were divided into nitrogen monoxide (NO) and Nitrogen dioxide (NO_2). Fine particulate matter ($PM_{2.5}$) was divided into 4 categories [52]: organic carbon (ORG); elemental carbon (EC); sulfate (SO_4) and nitrate (NO_3). The volatile organic compounds (VOC) were divided into 13 model species [51,53]: acetaldehyde (ALD); ketones (KET); benzene (TOL);

xylenes (XYL); propylene (OLT); dienes (OLI); ethylene (OL2); formaldehyde (HCHO); ethane (ETH); methanol (CH_3OH); ethanol (C_2H_5OH); propane (HC3) and alkanes (HC5).

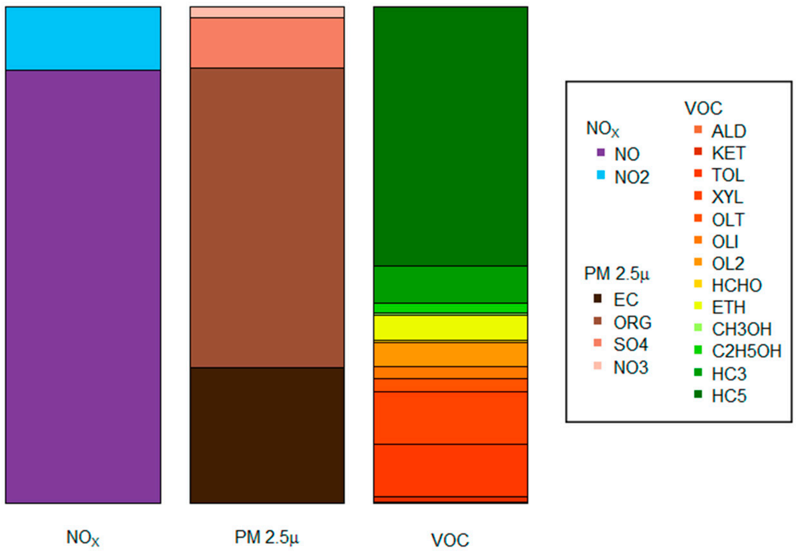

Figure S2. Mass fraction of nitrogen oxides (NO_x), fine particulate matter ($PM_{2.5}$) and non-methane volatile organic compounds (NMVOC).

2. Figures of Changes for Emission Scenarios

Figure S3 shows changes for surface O_3 on the 36 km resolution domain due to different emission scenarios (CLE, MIT, and MFR) for 2030, 2040 and 2050 considering the meteorological conditions of the RCP4.5 for 2020 for all cases. Each case was compared with a base case considering emissions of CLE for 2020 and meteorological conditions of RCP4.5 for 2020. Figure S4 shows the same comparison as in Figure S3, but for $PM_{2.5}$.

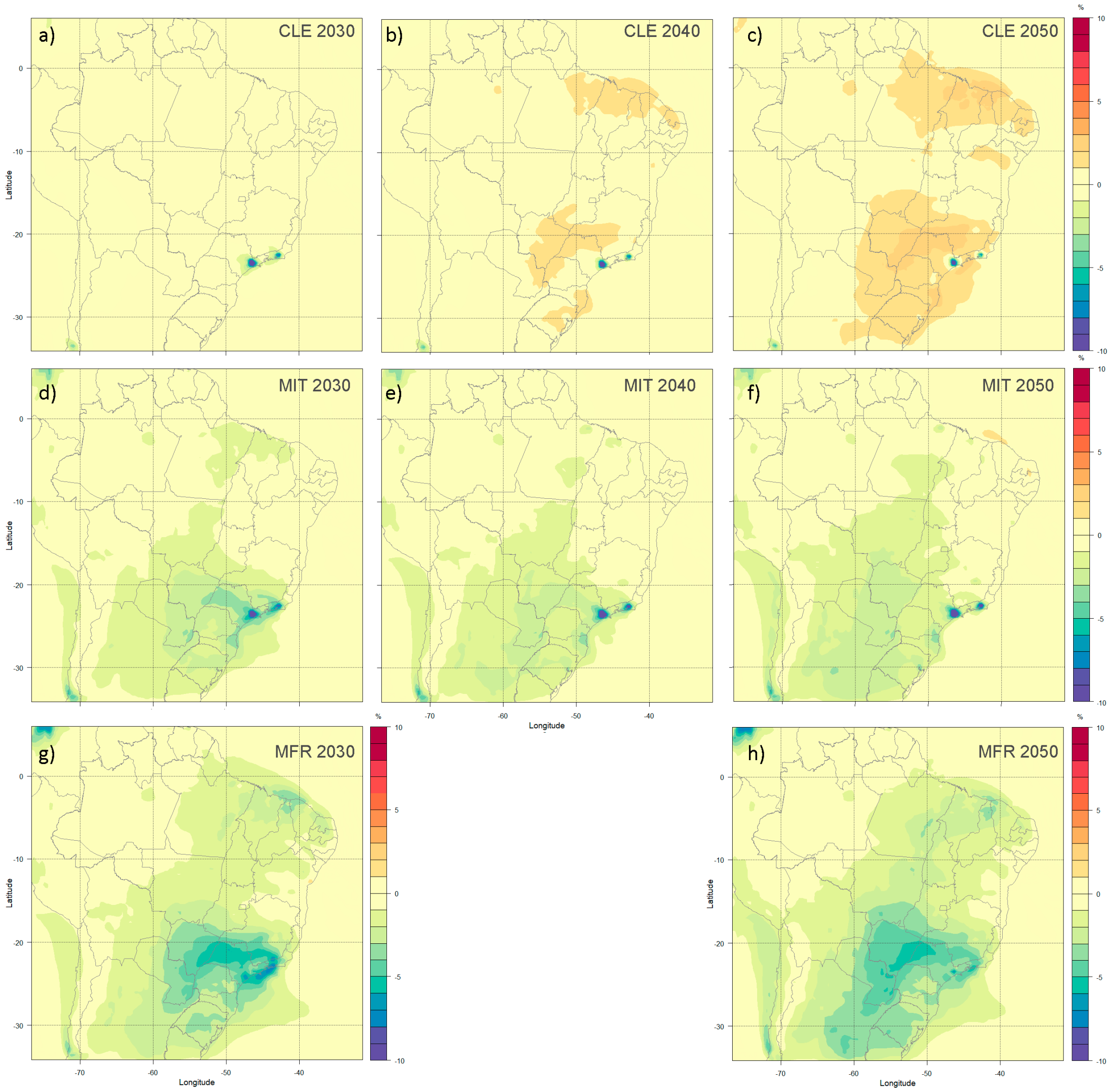

Figure S3. Percentage variation in surface O_3 on 36 km grid resolution domain due to emission changes: (**a**) CLE for 2030 (**b**) CLE emissions for 2040, (**c**) CLE emission for 2050, (**d**) MIT emission for 2030, (**e**) MIT emission for 2040, (**f**) MIT emission for 2050, (**g**) MFR emission for 2030 and (**h**) MFR emission for 2050.

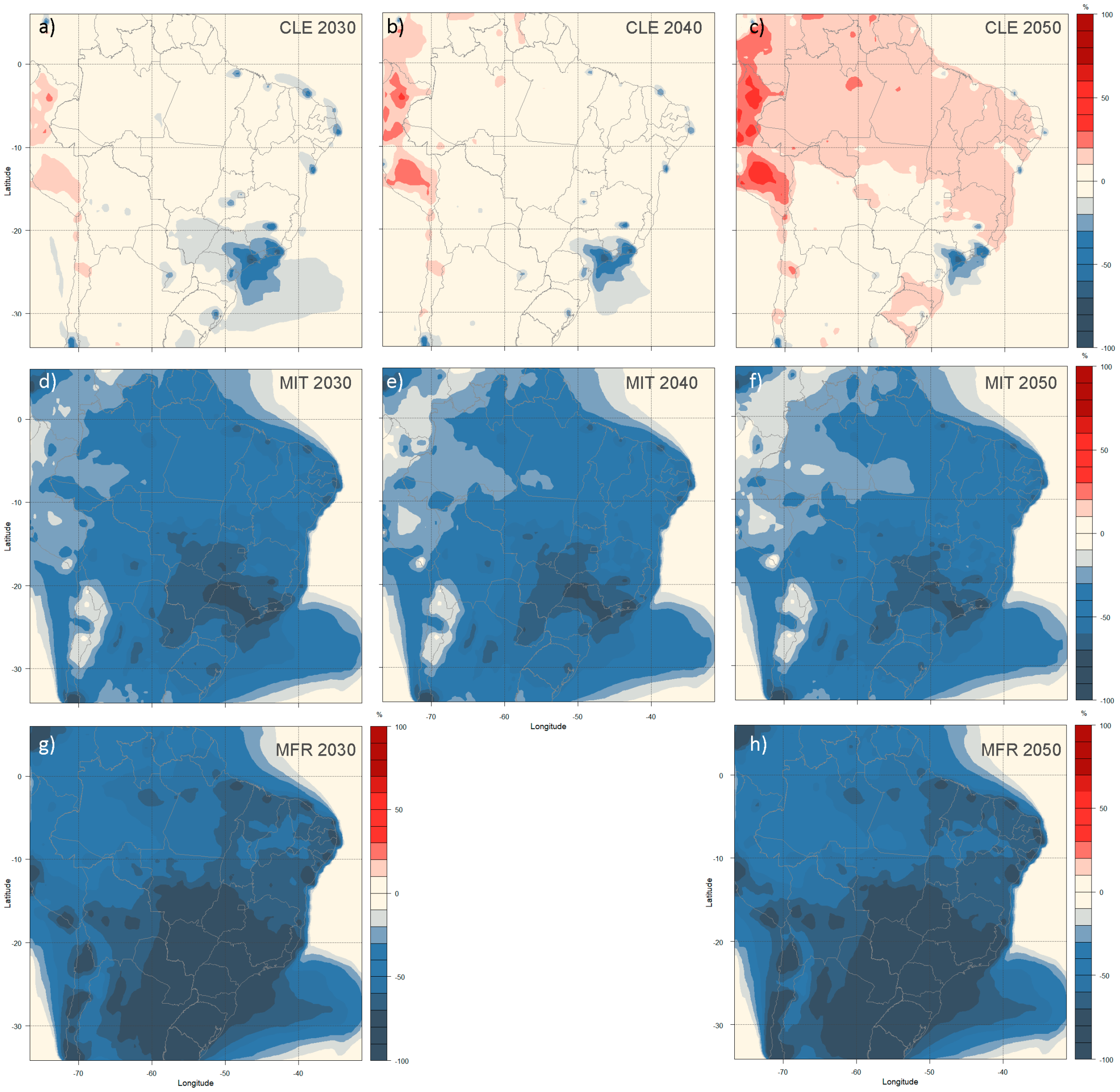

Figure S4. Percentage variation in surface $PM_{2.5}$ on 36 km grid resolution domain due to emission changes: (**a**) CLE for 2030 (**b**) CLE emissions for 2040, (**c**) CLE emission for 2050, (**d**) MIT emission for 2030, (**e**) MIT emission for 2040, (**f**) MIT emission for 2050, (**g**) MFR emission for 2030 and (**h**) MFR emission for 2050.

Figure S5 and Figure S6 shows the same comparisons of Figure S3 and Figure S4 (respectively) for the 9 km grid resolution domain.

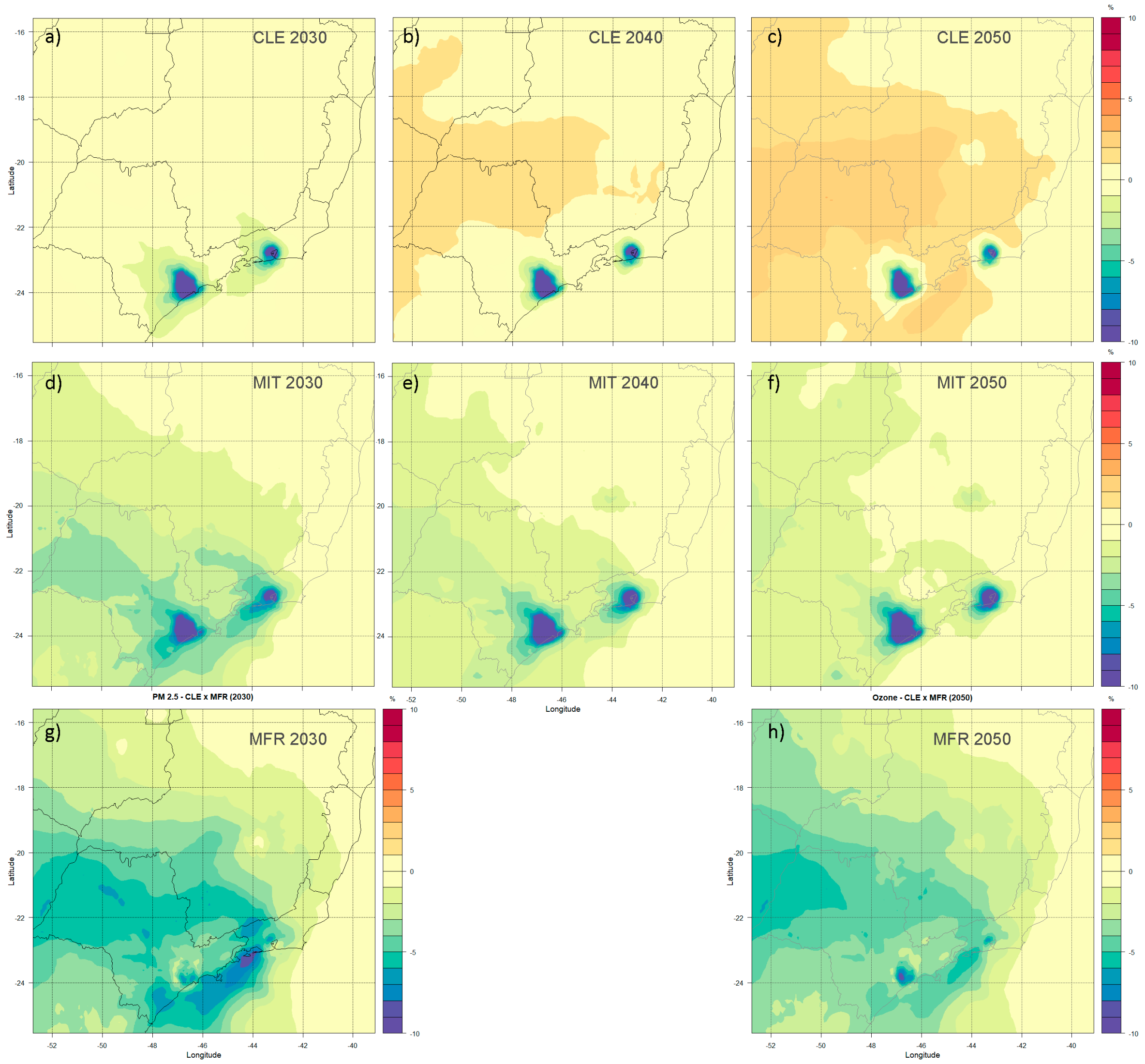

Figure S5. Percentage variation in surface O_3 on the 9 km grid resolution domain due to emission changes: (**a**) CLE for 2030 (**b**) CLE emissions for 2040, (**c**) CLE emission for 2050, (**d**) MIT emission for 2030, (**e**) MIT emission for 2040, (**f**) MIT emission for 2050, (**g**) MFR emission for 2030 and (**h**) MFR emission for 2050.

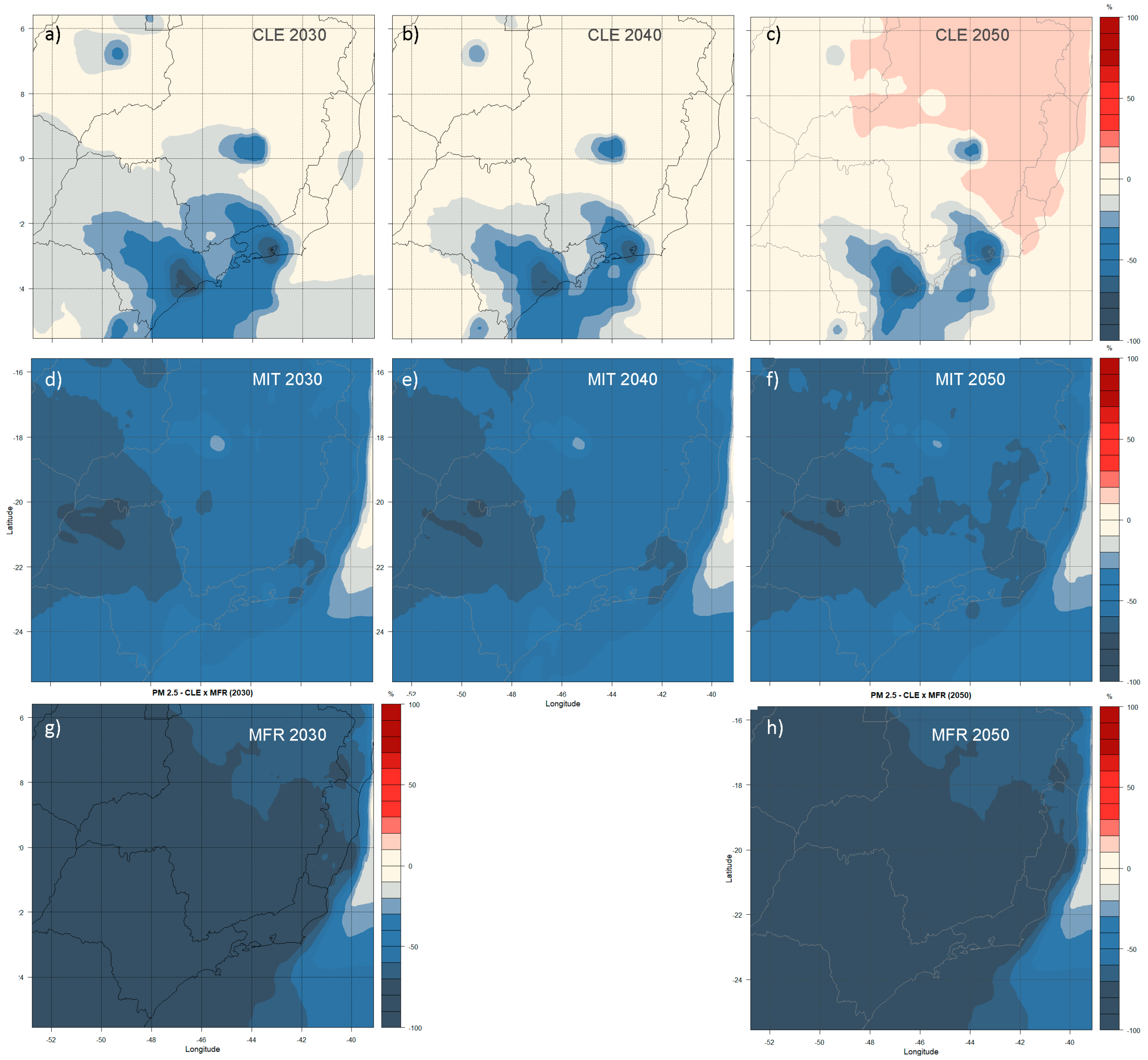

Figure S6. Percentage variation in surface $PM_{2.5}$ on the 9 km grid resolution domain due to emission changes: (**a**) CLE for 2030 (**b**) CLE emissions for 2040, (**c**) CLE emission for 2050, (**d**) MIT emission for 2030, (**e**) MIT emission for 2040, (**f**) MIT emission for 2050, (**g**) MFR emission for 2030 and (**h**) MFR emission for 2050.
